# A case series of Meckel’s diverticulum: usefulness of double-balloon enteroscopy for diagnosis

**DOI:** 10.1186/1471-230X-14-155

**Published:** 2014-08-30

**Authors:** Masashi Fukushima, Chiharu Kawanami, Satoko Inoue, Akihiko Okada, Yukihiro Imai, Tetsuro Inokuma

**Affiliations:** Departments of Gastroenterology, Kobe City Medical Center General Hospital, 2-1-1 Minatojimaminamimachi, Chuo-ku, Kobe Hyogo, 650-0047 Japan; Department of Gastroenterology, Otsu Red Cross Hospital, 1-1-35 Nagara, Otsu, Shiga, 520-8511 Japan; Department of Pathology, Kobe City Medical Center General Hospital, 2-1-1 Minatojimaminamimachi, Chuo-ku, Kobe, Hyogo 650-0047 Japan

**Keywords:** Meckel’s diverticulum, Double-balloon enteroscopy, Obscure gastrointestinal bleeding

## Abstract

**Background:**

Meckel’s diverticulum is a congenital anomaly of the gastrointestinal tract. About 98% of affected patients are asymptomatic. Small intestinal examination has become easier since the development of double-balloon enteroscopy. The present case series describes 10 patients with Meckel’s diverticulum in whom double-balloon enteroscopy was useful for diagnosis.

**Case presentation:**

Ten patients (8 men, 2 women) with Meckel’s diverticulum underwent double-balloon enteroscopy at Kobe City Medical Center General Hospital from May 2004 through May 2013. Their median age was 31.5 years (range, 14–83 years). Ten retrograde and two anterograde double-balloon enteroscopy procedures were performed. Double-balloon enteroscopy showed Meckel’s diverticulum in nine patients, but an inverted Meckel’s diverticulum was diagnosed as a lipoma in one patient. Meckel’s diverticulum was detected by iodinated contrast medium during anterograde double-balloon enteroscopy in one of the two patients who underwent this procedure. Meckel’s diverticulum was suspected using capsule endoscopy in one of two patients who underwent this procedure. Abdominal computed tomography was performed in all patients and revealed abnormalities in six, but Meckel’s diverticulum was suspected in only two. Technetium-99 m pertechnetate scintigraphy and a small bowel series were carried out in six patients, revealing Meckel’s diverticulum in one and three patients, respectively. Surgery was performed in eight patients, and endoscopic resection was carried out in one; the remaining patient was transferred to another hospital. Ulcer formation was found in or near Meckel’s diverticulum in eight patients.

**Conclusion:**

Compared with other modalities, double-balloon enteroscopy is excellent for the diagnosis of Meckel’s diverticulum because direct observation of both Meckel’s diverticulum and ulceration is possible. Double-balloon enteroscopy should be used complementarily to other less invasive examinations when needed to confirm or establish the diagnosis.

## Background

Meckel’s diverticulum (MD) is a congenital anomaly of the gastrointestinal tract. MD is usually located about 100 cm proximal to the ileocecal valve. Although MD is usually asymptomatic, about 2% of cases are symptomatic. Sixty percent of patients who develop complications are younger than 2 years old. Among patients with symptomatic MD, the male: female ratio is approximately 3:1 [1].

Observation of the small intestine has become easier since the development of double-balloon enteroscopy (DBE) and single-balloon enteroscopy. The number of reports of MD observed by DBE is increasing [2–4]. The present case series describes 10 patients with MD in whom DBE was useful for diagnosis.

## Case presentation

From May 2004 through May 2013, a total of 10 patients (8 men, 2 women) with MD underwent DBE at Kobe City Medical Center General Hospital. Their median age was 31.5 years (range, 14–83 years). Nine patients underwent esophagogastroduodenoscopy (EGD) before DBE. All patients underwent colonoscopy (CS) before DBE. The EN-450 T5/W and EN-450P5/20 gastrointestinal endoscopes (Fujifilm Corporation, Tokyo, Japan) were used in this case series. Ten retrograde and two anterograde DBEs were performed. All patients underwent conscious sedation with midazolam and pentazocine administered by an endoscopist. Patients who were scheduled to undergo retrograde DBE underwent bowel preparation using polyethylene glycol electrolyte solution (2 L). Two patients underwent capsule endoscopy (CE) (Pillcam SB and PillCam SB2; Given Imaging, Yokneam, Israel). Abdominal computed tomography (CT) was performed in all patients. Technetium-99 m (Tc-99 m) pertechnetate scintigraphy and a small bowel series were carried out in six patients.

**Figure 1:**
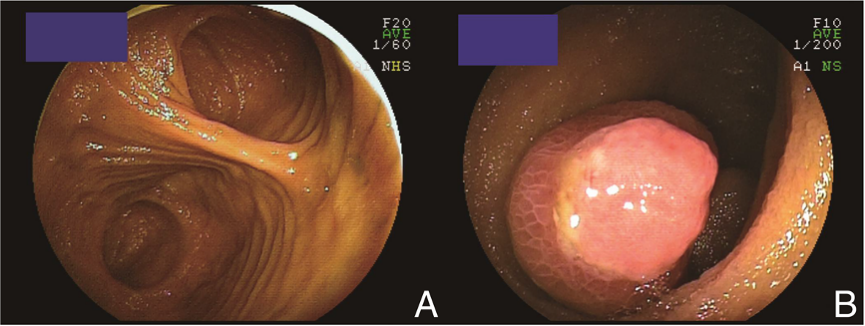
**Meckel’s diverticulum observed by double-balloon enteroscopy. A**: One lumen, located at the bottom of the screen, became a blind end (Case 8). **B**: Inverted Meckel’s diverticulum with ulceration (Case 10).

**Figure 2:**
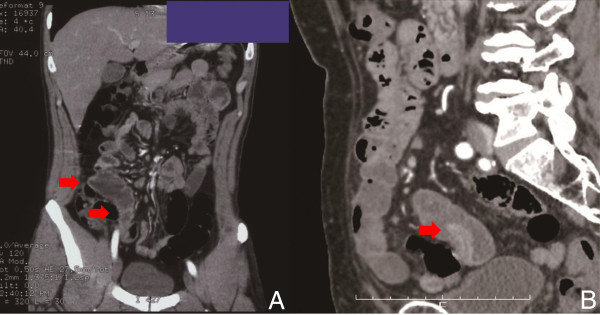
**CT findings of Meckel’s diverticulum. A**: Abdominal CT showed an extended part in the distal ileum. Continuity between the extended part and small intestine was unknown (Case 7). **B**: Abdominal CT revealed a polypoid lesion in the distal ileum. The peripheral branch of the ileocolic artery entered Meckel’s diverticulum. The polypoid lesion was regarded as Meckel’s diverticulum (Case 10).

**Figure 3:**
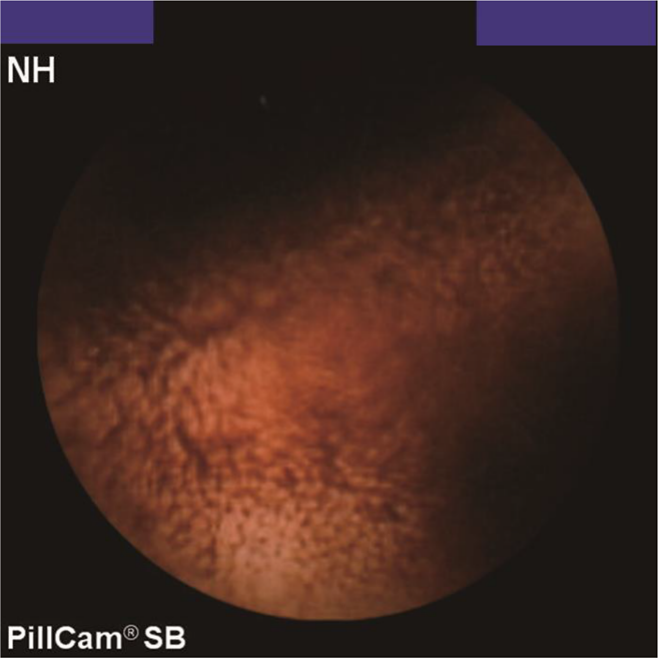
**Capsule endoscopy revealed bifurcation and hemorrhage of the intestinal tract, so Meckel’s diverticulum was suspected (Case 8).**

**Figure 4:**
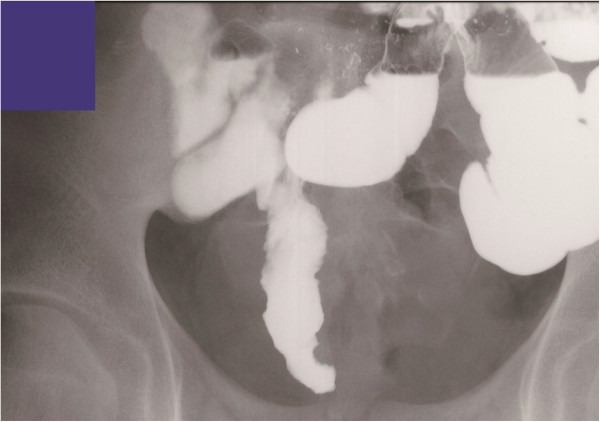
**Small bowel series detected Meckel’s diverticulum (Case 9).**

The clinical characteristics of patients who underwent DBE for MD are shown in Table 1. Overt gastrointestinal bleeding occurred in eight patients. Abdominal pain developed in three patients. DBE showed MD in nine patients (Figure 1A,B), but an inverted MD was diagnosed as a lipoma in Case 4. MD was detected by iodinated contrast medium during anterograde DBE in Case 7. Although CT revealed abnormalities in six patients, MD was suspected in only two (Figure 2A,B). Tc-99 m pertechnetate scintigraphy revealed MD in one patient (Neither cimetidine nor proton pump inhibitors were used in all cases before MD scan). MD was suspected using CE in one patient (Figure 3). A small bowel series revealed MD in three patients (Figure 4). Surgery was performed in eight patients, and endoscopic resection was carried out in one patient. One patient was transferred to another hospital. Ectopic gastric tissue was found in five patients, and ectopic pancreatic tissue was found in two patients. Ulcer formation was found in or near MD in eight patients. Symptoms associated with MD were not seen after the operation or endoscopic resection in all patients with the exception of Case 3.

**Table 1:** **Clinical characteristics of patients who underwent double-balloon enteroscopy for Meckel’s diverticulum**

Case	Age/Sex	Symptom		Abdominal CT	Tc-99m	CE	Double-balloon enteroscopy		Ulcer	Treatment	Ectopic tissue
			Small		pertechnetate		Anterograde	Retrograde			
			bowel series		scintigraphy						
1	26/M	GI bleeding	(4) Detected	(1) Not detected	(2) Detected	N/A	N/A	(3) Detected	(+)	Operation	Gastric tissue
2	21/F	GI bleeding	(3) Not detected	(1) Not detected	(2) Not detected	N/A	N/A	(4) Detected	(+)	Operation	None
3	14/M	Abd pain, vomiting	(2) Not detected	(1) Not detected	N/A	N/A	N/A	(3) Detected	(-)	Unknown	Unknown
4	58/F	GI bleeding, abd pain	N/A	(1) Polypoid lesion	N/A	N/A	N/A	(2) Detected	(+)	Endoscopic resection	Pancreatic tissue
5	40/M	Abd pain	(3) Detected	(1) Large cystic mass	(2) Not detected	N/A	N/A	(4) Detected	(+)	Operation	Gastric tissue
6	59/M	GI bleeding	N/A	(1) Extravasation	N/A	N/A	N/A	(2) Detected (twice)	(+)	Operation	Gastric tissue
7	29/M	GI bleeding	N/A	(1) Detected	N/A	(2) Not detected	(3) Not detected*	N/A	(+)	Operation	Gastric tissue
8	34/M	GI bleeding	(6) Not detected	(1) Not detected	(4) Not detected	(2) Detected	(3) Not detected	(5) Detected	(+)	Operation	None
9	16/M	GI bleeding, abd pain	(2) Detected	(1) Intussusception	(4) Not detected	N/A	N/A	(3) Detected	(-)	Operation	Gastric tissue
10	83/M	GI bleeding	N/A	(1) Detected	(2) Not detected	N/A	N/A	(3) Detected	(+)	Operation	Pancreatic tissue

Tc-99m, technetium-99m; CE, capsule endoscopy; N/A, not applicable; M, male; F, female; Abd, abdominal.

*Meckel’s diverticulum was identified using iodinated contrast medium through the scope.

The number in parentheses indicated the order of examination modality in inidividual case.

### Case 4

A 58-year-old woman was admitted to another hospital because of melena. Abdominal CT revealed a pedunculated, polypoid lesion with fat density in the ileum. The patient was transferred to our hospital for close examination. This polypoid lesion was diagnosed as a lipoma. Retrograde DBE was performed for the purpose of endoscopic resection. A pedunculated polyp was found in the distal ileum. Endoscopic resection was performed. Histologic findings identified an inverted MD with ectopic pancreatic tissue. No adverse events occurred, including perforation or hemorrhage [5].

### Case 5

A 40-year-old man presented to our hospital because the intermittent epigastralgia that he had experienced for 2 years had become stronger. Abdominal CT revealed a large cystic mass in the right lower abdominal quadrant. Retrograde DBE showed MD. He visited our emergency room because of strong abdominal pain in the right lower quadrant 1 month later. Acute appendicitis or Meckel’s diverticulitis was suspected. Emergent surgery was conducted because of the development of panperitonitis. Histological findings revealed gangrenous appendicitis with perforation and MD with ectopic gastric tissue. Meckel’s diverticulitis was not seen.

### Case 6

A 59-year-old man was transferred to our hospital because of hematochezia with hypovolemic shock. Emergent retrograde DBE was performed without bowel preparation, but we were not able to insert the endoscope ahead of the cecum because of massive stool and blood. Retrograde DBE after bowel preparation was performed again the next day. MD was detected, and Meckel’s diverticulectomy was performed.

### Case 7

A 29-year-old man was admitted to another hospital because of hematochezia and syncope. The patient was transferred to our hospital for close examination. Abdominal CT showed an extended part in the distal ileum. It was suspected that the extended part was MD because the continuity between the extended part and small intestine was unknown. Anterograde DBE was performed because of the potential inability to reach the lesion with a retrograde approach due to massive clots and blood. We reached the middle ileum but did not detect a hemorrhagic lesion. Because the endoscope could not be inserted any deeper, we used iodinated contrast medium through the scope while the balloons were inflated. MD was detected at about 20 cm on the anal side. Emergent Meckel’s diverticulectomy was performed.

### Case 8

A 34-year-old man was admitted to another hospital because of hematochezia. The patient was transferred to our hospital for examination and treatment. CE detected hemorrhage and a branch of the intestinal tract in the ileum; therefore, MD was suspected. Anterograde DBE was performed for the same reason as in Case 7. No abnormalities were found within the observed region. We used iodinated contrast medium through the scope while the balloons were inflated; however, no abnormalities were detected. The gastrointestinal bleeding stopped, so retrograde DBE was performed. MD was detected. Meckel’s diverticulectomy was performed.

### Case 9

A 16-year-old male patient was admitted to our hospital because of abdominal pain, vomiting, and hematochezia. Abdominal CT and ultrasound revealed a small bowel intussusception. A high enema was performed using physiological saline and contrast media. The intussusception was released by the high enema. Retrograde DBE revealed MD. Meckel’s diverticulectomy was performed.

### Case 10

An 83-year-old man visited another hospital because of melena and hematochezia. The patient was referred to our hospital for close examination. Abdominal CT showed a polypoid lesion in the distal ileum. An inverted MD was suspected. Retrograde DBE revealed an inverted MD. Its surface comprised normal intestinal mucosa. Meckel’s diverticulectomy was performed.

## Conclusions

**Figure 5:**
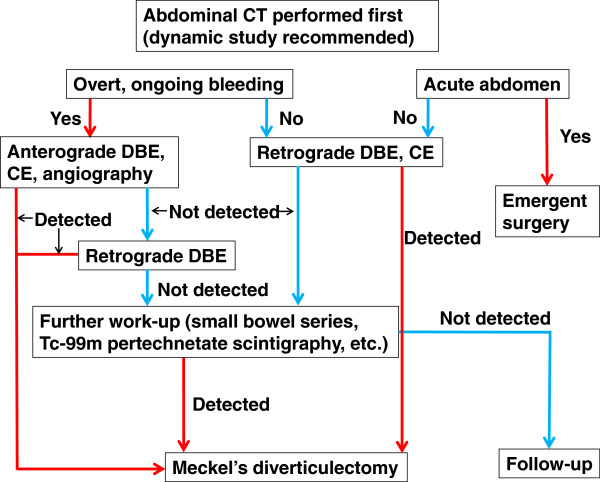
**Our hospital’s diagnostic flowchart of Meckel’s diverticulum.**

The area evaluated during small bowel examination has increased with the development of DBE, single-balloon enteroscopy, and CE. In the diagnosis of MD, DBE and single-balloon enteroscopy are useful especially because direct observation of MD and ulceration is possible. DBE and single-balloon enteroscopy also enable the performance of biopsy and endoscopic hemostasis. We directly observed MD using DBE in nine cases. MD is usually situated about 100 cm from the ileocecal valve, so retrograde DBE is particularly useful. A restriction of emergent retrograde DBE is that bowel preparation is required. In Case 7, we could not reach MD by anterograde DBE, but we identified MD using iodinated contrast medium through the scope. This method is useful when retrograde DBE is difficult because of massive hemorrhage and clots; however, this method is not always successful, as shown in Case 8. CE is also effective; however, the movement of the capsule is passive, making biopsy, insufflation, and endoscopic hemostasis impossible. The capsule could become trapped in MD [4]. CE may be more useful to determine the approach of DBE (peroral or anal). Whether DBE or CE is performed first depends on the current clinical situation. DBE should precede CE in an emergency. Abdominal CT, Tc-99 m pertechnetate scintigraphy, and small bowel series are also effective. Abdominal CT is particularly useful to detect bleeding, intussusception, and similar conditions. However, DBE is comparatively better able to identify MD as shown in the present report. Tc-99 m pertechnetate scintigraphy revealed MD in only one of six patients. If Tc-99 m pertechnetate scintigraphy results are negative, the presence of MD cannot be denied. Tc-99 m pertechnetate scintigraphy is a good diagnostic modality for children because of its minimal invasiveness. Our hospital’s diagnostic flowchart for MD is shown in Figure 5.

In Case 8, the histological findings indicated an inverted MD, but it was not inverted when DBE was performed. How inversion of MD occurs is unknown. It has been suggested that abnormal peristaltic movement caused by ulceration or ectopic tissue at the bottom of MD may cause it to invert [6]. In Cases 4, 8, 9, and 10 (and possibly Case 3), MD was inverted at least once. In Cases 4, 8, and 10, there was no ectopic gastric tissue, but ulceration of MD was observed. Endoscopic observation of the ulcers in MD provides important evidence of bleeding [4]. How ulcerations develop without ectopic gastric tissue is unknown. Chronic mechanical trauma or ischemia due to intermittent intussusception can be a cause [3, 7]. A persistent omphalomesenteric artery, which is a feeding artery of MD, is an end branch of the superior mesenteric artery and does not anastomose with other ileal branches. Therefore, this may provide another theory regarding ischemic ulceration of MD, whether inverted or not [8]. As shown in Case 9, an ulcer is not always seen in MD with ectopic gastric tissue. An ulcer may recover automatically; however, it is necessary to consider other factors as the cause of bleeding. In Case 9, mechanical stimulation and ischemia due to intussusception were considered to be the cause of bleeding, as in MD without ectopic gastric tissue.

Endoscopic resection of an inverted MD was carried out in Case 4 [5]. Adverse events did not occur, but perforation due to endoscopic resection has been reported [9]. Diagnosis of inverted MD is difficult; however, careful observation of the surface pattern of MD and abdominal CT lead to the diagnosis.

DBE and single-balloon enteroscopy are excellent for MD diagnosis; however, it is necessary to use other modalities complementarily. Symptomatic MD (gastrointestinal bleeding, abdominal pain, intussusception, and so on) necessitates surgery.

## Consent

Written informed consent was obtained from all patients for publication of this case report and any accompanying images. A copy of the written consent is available for review by the Series Editor of this journal.

## Abbreviations

MD: Meckel’s diverticulum
DBE: Double-balloon enteroscopy
EGD: Esophagogastroduodenoscopy
CS: Colonoscopy
CE: Capsule endoscopy
CT: Computed tomography
Tc-99 m: Technetium-99 m
Hb: Hemoglobin
M: Male
F: Female
Abd: Abdominal.
